# Supramolecular hydrogel microcapsules *via* cucurbit[8]uril host–guest interactions with triggered and UV-controlled molecular permeability†
†Electronic supplementary information (ESI) available. See DOI: 10.1039/c5sc01440a



**DOI:** 10.1039/c5sc01440a

**Published:** 2015-06-11

**Authors:** Ziyi Yu, Jing Zhang, Roger J. Coulston, Richard M. Parker, Frank Biedermann, Xin Liu, Oren A. Scherman, Chris Abell

**Affiliations:** a Department of Chemistry , University of Cambridge , Lensfield Road , Cambridge CB2 1EW , UK . Email: ca26@cam.ac.uk; b Melville Laboratory for Polymer Synthesis , Department of Chemistry , University of Cambridge , Lensfield Road , Cambridge CB2 1EW , UK . Email: oas23@cam.ac.uk

## Abstract

Host–guest assembly in droplet-based microfluidics opens a new avenue for fabricating supramolecular hydrogel microcapsules with high monodispersity and controlled functionality. In this paper, we demonstrate a single emulsion microdroplet platform to prepare microcapsules with supramolecular hydrogel skins from host molecule cucurbit[8]uril and guest polymer anthracene-functionalized hydroxyethyl cellulose. In contrast to construction of microcapsules from a droplet-in-droplet double emulsion, here the electrostatic attraction between charged polymer and surfactant facilitates formation of defined supramolecular hydrogel skins in a single emulsion. Furthermore, by taking advantage of dynamic interactions and the tunable cross-linked supramolecular hydrogel network, it is possible to prepare microcapsules with triggered and UV-controlled molecular permeability. These could be potentially used in a delivery system for *e.g.* agrochemicals, nutraceuticals or cosmetics.

## Abstract 2

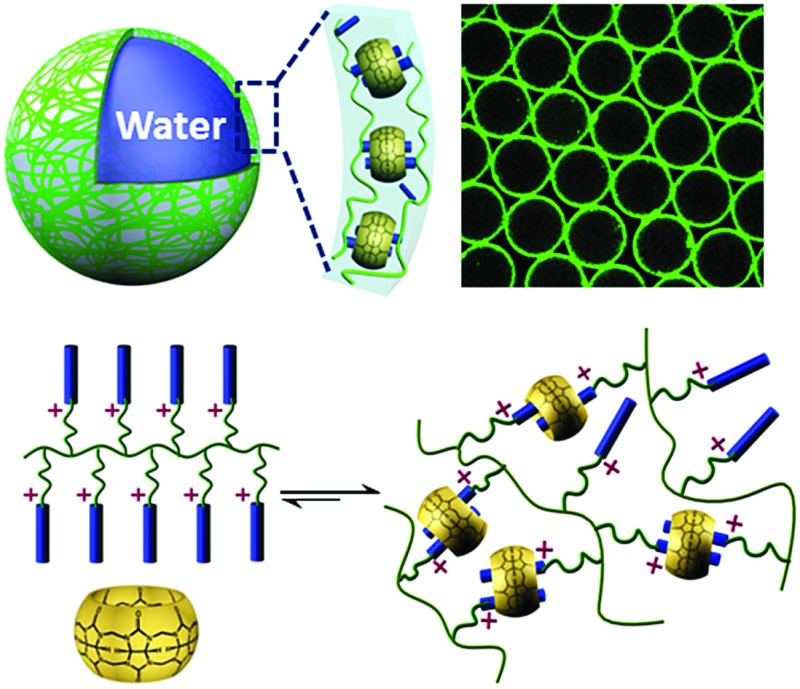
Host–guest assembly at the interface of microfluidic droplets offers a versatile strategy to construct supramolecular hydrogel microcapsules with “smart” cargo release.

## Introduction

There has been a significant growth in the study of microcapsules, no doubt inspired by their numerous applications in fields ranging from biotechnology and biopharmaceuticals, the food industry and even incorporation within electronic displays.^1–8^ Microcapsules have defined skins with a hollow structure which offers more space for cargo loading compared with solid microparticles.^9^ Recently, particular interest has been paid towards the design of “smart” microcapsules that are responsive to external stimuli, allowing release of their cargo in a controlled manner.^10–13^ Examples include formation of microcapsules with hydrogel skins that can be triggered to release drugs, cells, proteins, or volatile flavour components, upon the application of stimuli such as changes in light,^14,15^ redox,^16^ pH,^17^ or temperature.^18^ Stimuli-responsive hydrogels are three-dimensional chemical or physical cross-linked polymer networks that can swell or shrink in water, and release their payload in a controlled way.^19^ Recent contributions to construct hydrogel skins mostly rely on layer-by-layer assembly. Although powerful, the requirement for multiple immersion-centrifugation/filtration-washing steps render the preparation process both time and material intensive.^20–22^

Droplet microfluidics is a microchip-based emulsion technique which has been extensively applied over the past decade in the preparation of hydrogel microcapsules.^23–25^ The monodisperse nature of droplet production in a microfluidic device provides a unique reaction environment for the formation of microcapsules with very low polydispersity and high fabrication throughput, with efficient use of reagents.^26^ However, the reported microfluidic methods are mostly based on engulfment of templates by a hydrogel shell, generation of ‘droplet-in-droplet’ double or triple emulsions, followed by solidification of the outer layer of the droplets.^27,28^ Introduction of colloids to produce a Pickering emulsion, where colloids can adsorb to the water/oil interface, for subsequent polymeric cross-linking is an alternative way to construct colloidal hydrogel microcapsules.^29,30^ However, the hydrophobicity, shape, and size of the colloids must be carefully designed.

In this paper, we employ single emulsion droplet microfluidics to fabricate supramolecular hydrogel microcapsules using macrocyclic host–guest complexation, and further extend their stimulus responsive properties. Recent developments of supramolecular assembly have offered a framework to design advanced functional hydrogels.^31–33^ The supramolecular polymer hydrogels exhibit properties of traditional polymer hydrogels such as high water content and environmental sensitivity.^34,35^ Moreover, in view of the dynamic nature and reversibility of non-covalent interactions, the supramolecular hydrogels possess new functionality with potential for processability, recycling, and self-healing.^36–38^

**Fig. 1:**
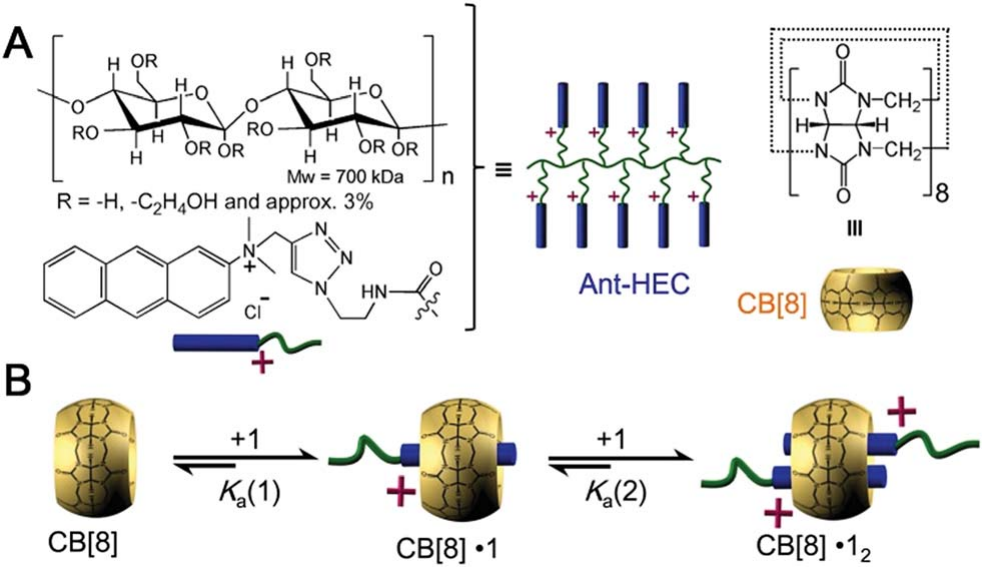
(A) Chemical structures of the components used in this study and their schematic representations: CB[8] and Ant-HEC. (B) Schematic illustration of the formation of homoternary complexes by accommodating two anthracene moieties in the cavity of CB[8].

Herein, we utilize supramolecular host–guest assembly between the macrocyclic host cucurbit[8]uril (CB[8]) and the guest of anthracene-functionalized hydroxyethyl cellulose (Ant-HEC) polymers (Fig. 1A) to fabricate supramolecular hydrogel skins for microcapsules. CB[8] is a macrocyclic molecule which is capable of accommodating up to two aromatic guest molecules simultaneously inside its cavity (Fig. 1B), to form either 1 : 2 CB[8]·(guest)_2_ homoternary complexes with monocationic guests or 1 : 1 : 1 heteroternary complex with both a dicationic and a neutral guest.^39–47^ The head-to-tail arrangement of two anthracene moieties within the cavity of CB[8] enables multiple non-covalent cross-links to form between adjacent anthracene-functionalized polymer chains,^44^ forming supramolecular polymer hydrogel skins at the water-in-oil interface of single microdroplets. This interaction can be disrupted by the introduction of a competitive guest, that displaces anthracene from within CB[8] giving a method to rupture the microcapsule and release encapsulated cargo.

Significantly, exposure of the pre-organized anthracene guests within the CB[8] host to UV light, stimulates photo-dimerization to generate a fully covalent microcapsule architecture. In this way, the supramolecular assembly can be used for fabrication of chemically stable, covalent microcapsules with different storage profiles compared to the dynamic non-covalent system. Adding the ability to determine the extent of covalency present in the microcapsule skin, we illustrated the use of precisely-controlled single emulsion microdroplet templating for tailored supramolecular cross-links. It is expected that this strategy would expands the potential of an already powerful microcapsule platform with new capabilities in the storage and delivery of a range of cargoes.

## Results and discussion

### Fabrication of supramolecular hydrogel microcapsules in droplet microfluidics

**Fig. 2:**
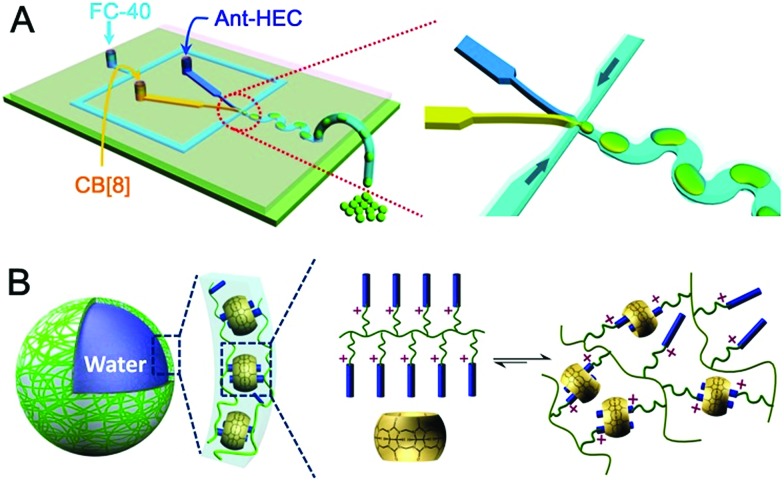
(A) Schematic representation of the preparation of supramolecular hydrogel microcapsules in droplet microfluidics. (B) Scheme showing the generation of supramolecular polymer hydrogel shells at the water/oil interface.

To fabricate supramolecular hydrogel microcapsules, monodispersed aqueous microdroplets containing a water soluble Ant-HEC and the macrocyclic host, CB[8], were generated in perfluorinated oil within a microfluidic device consisting of three inlets meeting at a single flow focus. This produced water-in-oil microdroplets in a single step at a frequency of 30 Hz (Fig. 2A). Flourinert FC-40 perfluorinated oil containing 3.0 wt% fluorous surfactant (XL-01-171) and 2.0 wt% Krytox 157FS-L was selected as the continuous phase, while the dispersed phase consisted of separate and individually controllable aqueous solutions of CB[8] (30 μM) and Ant-HEC containing 60 μM of the anthracene moiety. After injection, the two aqueous solutions met as a laminar co-flowing stream and on intersection with the perpendicularly flowing FC-40 continuous phase, were segmented into microdroplets. Finally, the microdroplets were passed through a winding channel to allow thorough mixing of the two reagents before collection (Fig. S1, ESI†). The concentrations of both Ant-HEC and CB[8] used were too low for significant complexation to occur in bulk solution, minimising cross-linking within the aqueous flow. However inside the microdroplet, the Ant-HEC concentrated at the water/oil microdroplet interface. This raises the local concentration of anthracene groups facilitating cross-linking by the CB[8] leading to formation of a supramolecular hydrogels, effectively templated on the spherical droplet interface (Fig. 2B).

**Fig. 3:**
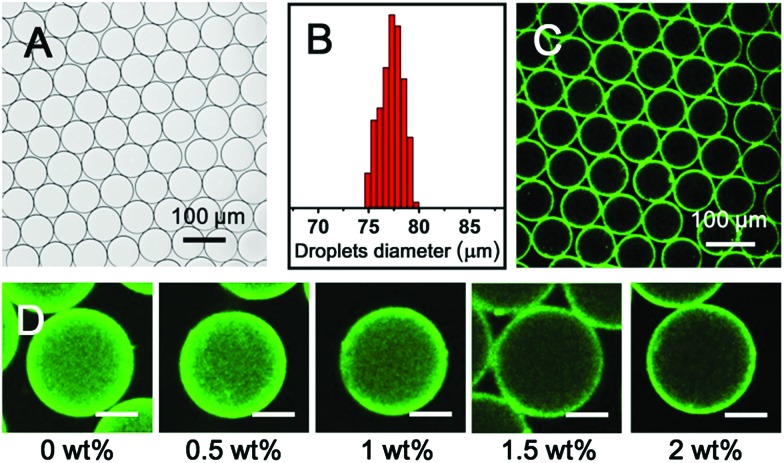
(A) Micrographs of microdroplets from aqueous solutions of 30 μM CB[8] and Ant-HEC containing 60 μM of the anthracene moiety. (B) Normalized distribution of microdroplet diameters, exhibiting a narrow size distribution (coefficient of variation 1.5%). (C) LSCM image of microdroplets illuminated with 405 nm laser and collected at emission range 480–520 nm. (D) LSCM images of microdroplets generated in stepwise in 0–2 wt% Krytox in FC-40 continuous phase (scale bars are all 30 μm).

Fig. 3A illustrates microdroplets prepared at a frequency of 30 Hz in the microfluidic device, with flow rates of the combined aqueous dispersed phase and FC-40 continuous phase of 30 μL h^–1^ and 60 μL h^–1^ respectively. The microdroplets exhibited a low level of polydispersity (mean diameter was 77 μm, with a coefficient of variation of 1.5%, Fig. 3B). As shown in Fig. 3C, laser scanning confocal microscopy (LSCM) showed that the fluorescence from the anthracene moieties in CB[8] is localised at the microdroplet interface, confirming the interfacial templating effect in the microdroplet. As it has been reported that fluorescence spectroscopy can track the π–π-stacking of anthracene-moieties within the cavity of the CB[8] host,^44^ the quantitative fluorescence in single microdroplets was studied with a varying molar ratio between CB[8] and anthracene (Fig. S2, ESI†). In the absence of CB[8], the fluorescence from microdroplets is mainly distributed in the blue waveband because of the single anthracene units. Upon increasing CB[8] concentration, green emission increases (489–531 nm) with corresponding reduction in blue emission (417–477 nm), attributed to π–π-stacking of anthracene within CB[8]. No further change in the fluorescence spectrum was observed upon increasing CB[8] concentration above 1.5 equivalents.

The driving force for formation of CB[8]·(Ant-HEC)_2_ supramolecular hydrogels to the microdroplet interface was attributed to an electrostatic attraction between the positive-charged Ant-HEC and the carboxylate on the Krytox surfactant.^48^ To illustrate this control over capsule formation, the Krytox concentration in the continuous oil phase was increased step-wise from 0–2 wt%. As shown in Fig. 3D, in the absence of Krytox, fluorescence was found to be uniform throughout the microdroplet, indicative of little preference for the microdroplet interface with capsule formation unlikely. In contrast, with increasing concentration of Krytox from 0.5 wt% to 2 wt%, the intensity of the fluorescence gradually increases at the interface of the microdroplets at the expense of the bulk droplet volume, until at 1.5 wt% or greater fluorescence appears to be exclusively originating from the water/oil interface. In this way, the electrostatic templating of Ant-HEC at the droplet interface leads to the crucial localised increase in concentration necessary for CB[8] to cross-link adjacent polymer chains, ultimately leads to formation of the supramolecular hydrogels skin.

**Fig. 4:**
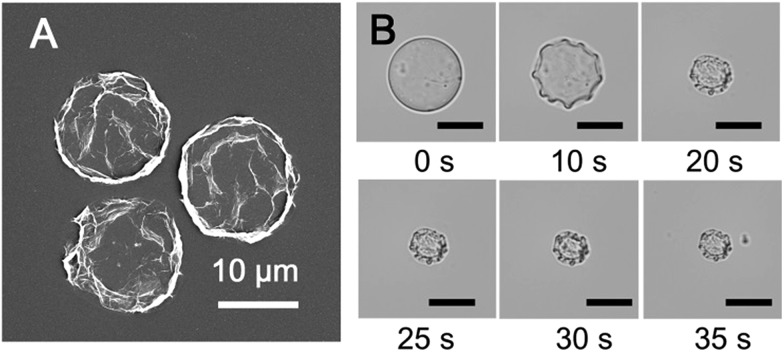
(A) SEM image of dried microcapsules. (B) Micrographs of the microcapsule drying process, resulting in a collapsed structure (scale bars are all 20 μm).

The microdroplets were collected and air dried, forming stable dehydrated microcapsule structures that can be stored (Fig. S3, ESI†) and studied by scanning electron microscopy (SEM). It was found that the hollow microcapsules collapsed upon evaporation of the aqueous core due to a lack of internal support, with creases and folds clearly visible on the supramolecular cross-linked polymer surface (Fig. 4A). This deformation and collapse of the microdroplet takes place over seconds, as illustrated in Fig. 4B, where the microcapsule becomes smaller as the water evaporates. This clearly demonstrates that a supramolecular hydrogel microcapsule skin has formed at the interface significantly prior to complete dehydration.

### Supramolecular hydrogel microcapsules with triggered or UV-controlled permeability

**Fig. 5:**
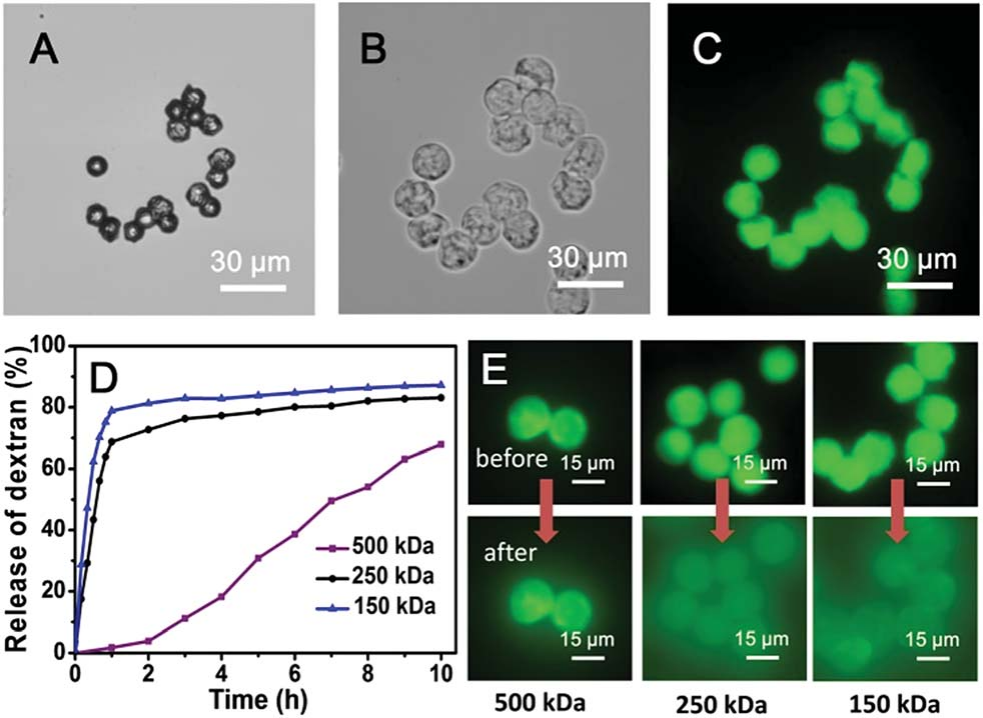
Micrographs of dried microcapsules containing 500 kDa FITC-dextran (A) before and (B) after rehydration. (C) Corresponding fluorescence micrograph of the rehydrated microcapsules containing 500 kDa FITC-dextran. (D) Release profiles of FITC-dextran from microcapsules as a function of the rehydration time. (E) Series of fluorescence micrographs of microcapsules loaded with different molecular weight FITC-dextran before and after 2 hours rehydration.

The simultaneous delivery of the capsule-forming components and aqueous-soluble cargo during microdroplet formation enables facile encapsulation of cargoes within the microcapsule. To investigate the potential utility of these microcapsules for the storage and release of cargoes, their encapsulation behaviour was studied using a family of fluorescein isothiocyanate-dextrans (FITC-dextran) as molecular probes. After rehydrating FITC-dextran-loaded microcapsules in water, they were studied by fluorescence microscopy (excitation at 470–495 nm and detection above 520 nm to avoid overlap with the fluorescence of the anthracene moiety). Upon rehydration in water, the size of the microcapsules encapsulating FITC-dextran (500 kDa) approximately doubled (Fig. 5A and B) due to osmotic pressure driven by the hydrophilic cargo. It is noteworthy that the microcapsules which were comprised of water soluble Ant-HEC polymers and CB[8], displayed a robust shell in water and successfully held FITC-dextran in their interior (Fig. 5C). In contrast, microcapsules prepared without CB[8] dissociated into small fragments on exposure to water, immediately releasing encapsulated cargo (Fig. S4, ESI†). This observation supports the interpretation that CB[8] is acting as a supramolecular “handcuff” to link two anthracene moieties, thereby preventing the dissociation of the three-dimensional hydrogel network in the microcapsule skin.

To evaluate the molecular permeability of the supramolecular hydrogel microcapsules, the release of FITC-dextran cargoes of different molecular weights was studied over time. The 150 kDa and 250 kDa FITC-dextran were released rapidly and quantitatively, with 80% and 70% release, respectively, of the total loading within the first hour (Fig. 5D). In contrast, the 500 kDa FITC-dextran loaded microcapsules showed slow release, 2% in the first hour, increasing up to 70% over 10 hours. The fluorescence micrographs (Fig. 5E) clearly show differential FITC-dextran release into the surrounding solution after 2 hours of rehydration. The 500 kDa FITC-dextran has a Stoke's radius of around 14.7 nm,^49^ thought to be close to the pore size of the microcapsule skin and therefore not able to readily diffuse out of the microcapsule.

**Fig. 6:**
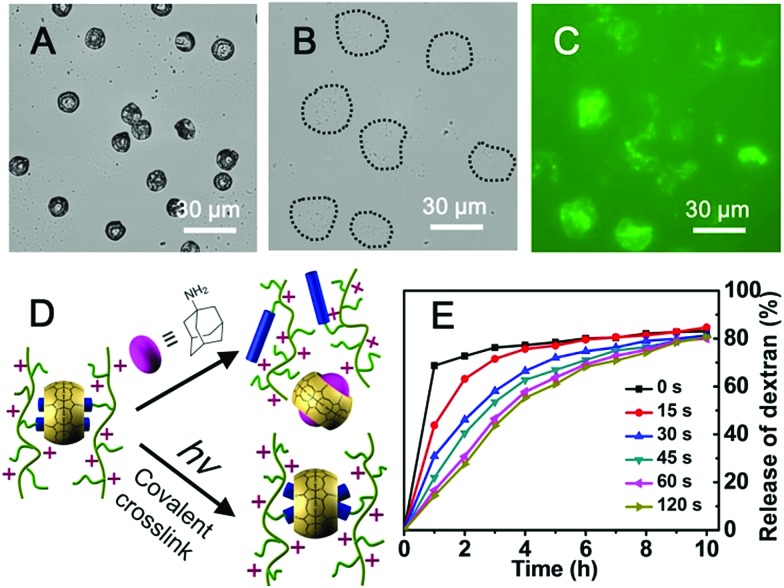
Micrographs of dried microcapsules containing 500 kDa FITC-dextran (A) before and (B) after rehydration in 1 mM aqueous ADA solution. (C) Corresponding fluorescence micrograph of 500 kDa FITC-dextran loaded microcapsules after rehydration in ADA. (D) Schematic representation of the disassembly of homoternary complexes in the absence of ADA molecules and the [4 + 4]-photodimerisation of anthracene groups in the CB[8] cavity. (E) FITC-dextran (250 kDa) release profiles from microcapsules as a function of the UV irradiation time.

The cargo in the CB[8]·(Ant-HEC)_2_ microcapsules can be released by treatment with a higher affinity ligand for CB[8] such as 1-adamantylamine (ADA, Fig. 6D).^50^ On exposure of microcapsules to an aqueous solution of ADA (1 mM), hydrated microcapsules undergo rupture after 3 minutes (Fig. 6A and B) accompanied by immediate dispersion of FITC-dextran cargo (Fig. 6C). This triggered release is attributed to the disassembly of the CB[8]·(Ant-HEC)_2_ hydrogel complexes.

Not only do CB[8]·(Ant-HEC)_2_ microcapsules offer triggered release of their cargo, their chemistry also allows for the formation of progressively covalently crosslinked hydrogels with the ability to control the molecular permeability of the microcapsules. This is achieved by taking advantage of the [4 + 4]-photo-dimerization of the two anthracene moieties pre-organized as guests in the CB[8] (Fig. 6D and S5, ESI†). Fig. 6E shows the release profile for microcapsules loaded with 250 kDa FITC-dextran as a function of the UV exposure time post-capsule formation. As the duration of UV irradiation increased, the rate and amount of FITC-dextran dispersed from the microcapsules was significantly reduced. Irradiation for as little as 30 s resulted in a reduction in cargo release from 70% to 30% in one hour. Increasing the UV irradiation time to 60 s again further slowed the loss of cargo, with 30% release now taking 2 hours. Extending the UV irradiation to 120 s had little additional effect, suggesting that the photo-cross-linking of dimerized anthracene units had neared saturation.

## Conclusions

We have demonstrated a host–guest assembly approach to prepare supramolecular hydrogel microcapsules through a homoternary complex from CB[8] and Ant-HEC polymers in microfluidic droplets. The electrostatically-directed attraction between the supramolecular polymer in water and the surfactant in the surrounding oil facilitates fabrication of defined supramolecular hydrogel skins from a single emulsion. Disruption of the supramolecular interaction leads to rupture of the hydrogel network, achieving triggered molecular permeability. In contrast, irradiation with UV light resulting in [4 + 4]-photo-dimerization of the anthracene guests. This converts the supramolecular hydrogel network from a non-covalent to covalent structure post-fabrication, offering a mechanism to control the permeability of the microcapsule. This increases the range of potential applications for these self-assembled hydrogel microcapsules with dramatically different architectures produced from small changes in simple parameters during fabrication or with post-processing.
