# Supplementary material for: Supramolecular hydrogel microcapsules via cucurbit[8]uril host–guest interactions with triggered and UV-controlled molecular permeability
Source: Chem Sci. 2015 Jun 11;6(8):4929–33. doi: 10.1039/c5sc01440a (PMC5504465; doi:10.1039/c5sc01440a)
Supplement: Supplementary file 1 [file SC-006-C5SC01440A-s001.pdf]

Electronic Supplementary Material (ESI) for Chemical Science.
This journal is © The Royal Society of Chemistry 2015

Electronic Supplementary Information

Supramolecular hydrogel microcapsules *via* cucurbit[8]uril host-guest interactions with triggered and UV-controlled molecular permeability †

Ziyi Yu, [a] Jing Zhang, [a] Roger J. Coulston,[b] Richard M. Parker, [a] Frank Biedermann,[b] Xin Liu, [a] Oren A. Scherman[b,]* and Chris Abell[a,]*

[a] Department of Chemistry, University of Cambridge, Lensfield Road, Cambridge CB2 1EW, United Kingdom E-mail: ca26@cam.ac.uk

[b] Melville Laboratory for Polymer Synthesis, Department of Chemistry, University of Cambridge, Lensfield Road, Cambridge CB2 1EW, United Kingdom E-mail: oas23@cam.ac.uk

Materials & Methods

General

Images of droplet formation were obtained using a Phantom v7.2 camera attached to an Olympus IX71 inverted microscope. Laser scanning confocal microscope (LSCM) measurements were carried out using a Leica TCS SP5 confocal microscope attached with a 10× objective. Samples were illuminated with 405 nm laser for exciting the 1:2 Cucurbit[8]uril (CB[8])·anthracene-functionalized hydroxyethyl cellulose (Ant-HEC) ternary complexes at 480-520 nm in microdroplets. Microscopic images and fluorescence images were obtained using an Olympus IX81 inverted optical microscope coupled with a camera of Andor Technology EMCCD iXonEM+ DU 897. The quantitative fluorescence emission measurement of single microdroplet containing Ant-HEC with CB[8] was measured by a photomultiplier tube (H8249-102, Hamamatsu) when the microdroplet pass through a JDSU 150 mW 355 nm UV DPSS laser, with 510/42 filter (Semrock) and 447/60 filter (Semrock) to split the emission light. To image the fluorescence of FITC-dextran, a mercury lamp was installed for wide-spectrum illumination with FITC filters and dichroics fitted to separate the fluorescence excitation and emission light. Scanning electron microscopy (SEM) measurements were made and images recorded using a Leo 1530 variable pressure SEM with InLens detector. All starting materials were purchased from Sigma Aldrich and used as received unless stated otherwise. All aqueous solutions were made in deionized water treated with a Milli-Q^{TM} reagent system ensuring a resistivity of >15 MΩ cm^{-1}.

Synthesis

Synthesis of Cucurbit[8]uril (CB[8])

CB[8] was synthesised from glycoluril with formaldehyde by a basic procedures published by Day and Kim,[1, 2] further isolation and purification were carried out by previously reported method.[3]

Synthesis of anthracene functionalized hydroxyethyl cellulose (Ant-HEC) involved the following steps

(i) Synthesis of N, N-dimethylanthracen-2-amine

N, N-dimethylanthracen-2-amine was obtained via reductive amination from 2-amino-anthracene in analogy to literature procedures[3]. To a stirred slurry of 2-amino-anthracene (1.0 g, 5 mmol) and 4 mL (50 mmol) of 37% aqueous paraformaldehyde in 100 mL acetonitrile was added 950 mg (15 mmol) of sodium cyanoborohydride. Glacial acetic acid (0.5 mL) was added

over 10 min, and the reaction was stirred at room temperature for 2 h. An additional 0.5 mL of glacial acetic acid was added, and stirring was continued for another 30 min. The reaction mixture was poured into 150 mL of diethyl ether and then washed with three 20 ml portions of 1 N KOH and one 20-mL portion of brine. The ether solution was dried over magnesium sulfate and the solvent was evaporated under reduced pressure to yield the title compound in 95% yield. The crude material was of sufficient purity (>90% by NMR) for the following steps and not further purified.

(ii) Synthesis of N, N-dimethyl-N-(prop-2-yn-1-yl)anthracen-2-aminium chloride

To a solution of N, N-dimethylanthracen-2-amine (0.1 g, 0.5 mmol) in 20 mL acetone was added propagyl bromide (0.25 ml, 80 wt% solution in toluene, 2.5 mmol) and the reaction mixture was stirred under light protection for 7 days at room temperature. The resulting precipitate was collected by suction filtration and washed with diethyl ether to yield the NMR-pure product as its bromide salt. Counter-ion exchange to chloride was achieved by stirring and sonicating of an aqueous solution (ca 20 mL) of the product over freshly precipitated silver chloride (20 equiv. per bromide counter ion) for several hours. The silver salt was filtered off and the filter cake was washed with water. The combined aqueous solution was freeze-dried to yield the title compound as a yellowish solid (71 mg, 58%).

(iii) Synthesis of N3-HEC

Hydroxyethyl cellulose, (2.0 g, Mw = 700,000 g/mol) was dissolved in 150 mL N-methyl-2-pyrrolidone (NMP) at 110 ^{o}C with stirring. The solution was allowed to cool to room temperature and 1-chloro-3-isocyanatopropane (0.1 mL) and one drop of dibutyl tin dilaurate (TDL) was added. The reaction was stirred for 24 h at room temperature and then sodium azide (0.1 g) was added. The reaction mixture was heated to 80 ^{o}C and stirred for another 24 h. Upon cooling to room temperature and addition of 1.5 L acetone, the polymer precipitated and was collected by suction filtration. The solid was redissolved in a minimal amount of water and reprecipitated from acetone. The filter cake was washed with copious amounts of acetone and dried in the vacuum oven at 40 ^{o}C overnight to yield the title compound in 90% yield.

(iv) Synthesis of Ant-HEC

Azido functionalised hydroxyethyl cellulose, N3-HEC, (0.2 g, Mw = 700,000 g/mol) and N, N-dimethyl-N-(prop-2-yn-1-yl)anthracen-2-aminium chloride (40 mg) were dissolved under light protection in 200 mL water and degassed with nitrogen. To this solution was as added a degassed and sonicated (5 min, 42 kHz) solution of copper sulfate (1.5 mg, 0.001 mmol) and sodium ascorbate (1.8 mg, 0.001 mmol) in 5 mL water. The resulting mixture was stirred at room temperature for 24 h under light protection. The solvent was reduced to approx 25 mL under reduced pressure and 250 mL acetone was added. All work-up steps were performed under light protection. The precipitate was collected by suction filtration and washed with acetone. The solid was redissolved in 10 mL water and dialysed (regenerated cellulose membrane from Spectrum Labs with Mw (cutoff) = 3500 Da) against a 0.1% brine solution (3.5 L) and then against water for 48 h in the darkness. After freeze drying, the title compound was obtained in 85% yield (0.17 g) as a fluffy yellowish solid. From the absorbance at 254 nm of a 60 μg/mL stock solution, a polymer loading of approx. 80 μmol of the anthracene side chains per gram of polymer was calculated which equals approx. 30 mg of the anthracene moiety per gram of polymer.

<u>Microfluidic device fabrication</u>

The microfluidic device was produced via soft lithography by pouring poly(dimethylsiloxane) (PDMS) along with crosslinker (Sylgard 184 elastomer kit, Dow Corning, pre-polymer : crosslinker = 10 : 1) onto a silicon wafer patterned with SU-8 photoresist.[5, 6] The PDMS was allowed to solidify at 70 °C overnight before it was peeled off, while inlets and outlets were generated using a biopsy punch. The enclosed microfluidic channels were formed by attaching the moulded PDMS replica onto microscope slides after exposure to oxygen plasma for 8 s in a Femto plasma cleaner. To render the devices hydrophobic and suitable for water-in-oil emulsification, 200:1 Novec HFE-7500 (3M) Engineered Fluid oil and trichloro(1H,1H,2H,2H-perfluorooctyl)silane was injected into the microchannels of sealed devices, allowed them to coat on the surface of microchannels at 70 °C overnight.

<u>Preparation of microcapsules</u>

To generate water-in-oil microdroplets, three different liquids were injected into a microfluidic device, by three syringe pumps (PHD, Harvard Apparatus) with controlled flow rates. Flourinert FC-40 (3M) containing a 3 wt% fluorous surfactant (XL-01-171, kind gift from Dr Xin Li, Sphere Fluidics Ltd) and 2 wt% Krytox® 157FS (Dupont) was used as the continuous phase. One discontinuous aqueous phase was prepared by dissolving CB [8] in water, and another discontinuous phase was prepared by dissolving Ant-HEC in water. The continuous phase and both discontinuous phase solutions were loaded into three 1 mL syringes respectively before connecting to the microfluidic chip. Syringes with needles were mounted on syringe pumps and fitted with polyethylene tubing, while the other end of the tubing was inserted into the appropriate inlets of a microfluidic chip. Microdroplets formation was initiated as Flourinert FC-40 was first pumped into the device at the rate of 60 μL/h to fill the appropriate channels. The aqueous dispersed phase was then pumped into the device at 30 μL/h. In a typical experiment, the concentrations of Ant-HEC and CB[8] were 60 μM and 30 μM, respectively. After formation, microdroplets were either collected in the PDMS reservoir downstream or transferred to a glass bottom dish. Upon collection, droplets were allowed to dehydrate over 5 hours for the complete formation of isolated microcapsules. The diameter of droplets was meatured when the deformation begins, which defined as the diameter of prepared microcapsules. To generate FITC-dextran loaded microcapsules, FITC-dextran was directly mixed with the aqueous Ant-HEC solution and then pumped into the microfluidic device. The final concentration of FITC-dextran was 0.4 mg/mL.

<u>Molecular permeability experiments</u>

To investigate the molecular permeability of FITC-dextran, supramolecular microcapsules were fabricated containing FITC-dextran with molecule weight of 500 kDa, 250 kDa, and 500 kDa, respectively. After drying on a glass bottom dish, the prepared microcapsules were washed with HFE-7500 three times to remove the residual surfactants. The glass bottom dish was sealed with parafilm and mounted on the fluorescence microscope after a few drops of water were applied over the dried microcapsules. The fluorescence images were taken every 1 hour for 10 hours. For the quantitative analysis of the release of FITC-dextran, average fluorescence intensities were estimated in two regions of interest in a fluorescence image, one covering a rehydrated microcapsule, and the other one covering the whole background in the image. A temporal course of these two values was estimated from the stack of images by Image J.

To investigate the triggered molecular permeability of FITC-dextran, microcapsules were fabricated containing FITC-dextran (500 kDa) and dried on glass bottom dish as described

previously. After washed with HFE-7500 three times, a few drops of aqueous 1-aminoadamantane (ADA) solution (1 mM) were applied over the dried microcapsules. The glass bottom dish was sealed with parafilm and mounted on the fluorescence microscope. The fluorescence images were taken every 1 hour for 10 hours.

To demonstrate the UV-controlled molecular permeability of FITC-dextran, microcapsules were fabricated containing FITC-dextran (250 kDa) and dried on glass bottom dish as described previously. After washing with HFE-7500 three times, the prepared microcapsules were exposed to a UV light source using excitation filter cube of an Olympus IX81 microscope equipped with a 100 W mercury lamp (365 nm, USH-1030L, USHIO Inc.). The exposure times of different samples were 0 s, 15 s, 30 s, 45 s, 60s, and 120s, respectively. Then the glass bottom dish was sealed with parafilm and mounted on the fluorescence microscope after a few drops of water were applied over the dried microcapsules. The fluorescence images were taken every 1 hour for 10 hours.

References

1 A. Day, A. P. Arnold, R. J. Blanch and B. Snushall, *J. Org. Chem.*, 2001, **66**, 8094-8100.

2 J. Kim, I.-S. Jung, S.-Y. Kim, E. Lee, J.-K. Kang, S. Sakamoto, K. Yamaguchi and K. Kim, *J. Am. Chem. Soc.*, 2000, **122**, 540-541.

3 D. Jiao, N. Zhao and O. A. Scherman, *Chem. Commun.*, 2010, **46**, 2007-2009.

4 R. F. Borch and A. I. Hassid, *J. Org. Chem*, 1972, **37**, 1673-1674.

5 D. Qin, Y. Xia and G. M. Whitesides, *Adv. Mater.*, 1996, **8**, 917-919.

6 D. C. Duffy, J. C. McDonald, O. J. A. Schueller and G. M. Whitesides, *Anal. Chem.*, 1998, **70**, 4974-4984

Supplementary Figures

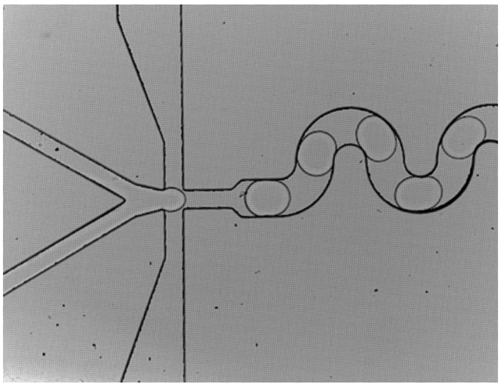

Fig. S1. Microscopic image of the flow-focusing microfluidic device and a wiggled channel for rapid mixing of CB[8] and Ant-HEC online.

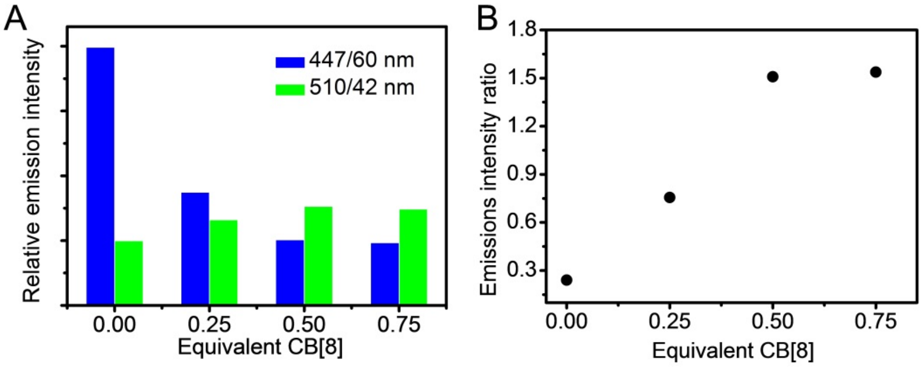

Fig. S2. (A) Fluorescence emission intensity in 417-477 nm and 489-531 nm of single microdroplet containing Ant-HEC upon addition of CB[8]. (B) The fluorescence emission intensity ratio between 417-477 nm and 489-531 nm of single microdroplet containing Ant-HEC upon addition of CB[8].

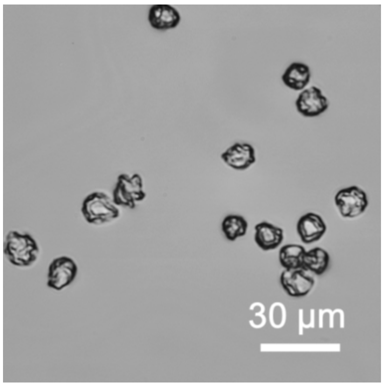

Figure S3 Microscopic image of prepared individual CB[8]/Ant-HEC stable microcapsules.

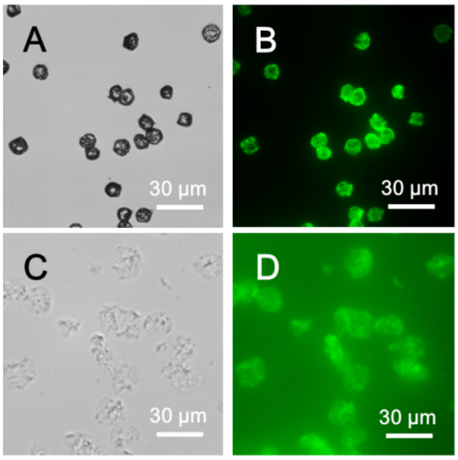

Fig. S4 Micrographs of dried Ant-HEC microcapsules containing 500 kDa FITC-dextran (A) before and (C) after rehydration in water. Fluorescence micrographs of rehydrated CB[8]/Ant-HEC microcapsules containing 500 kDa FITC-dextran (B) before and (C) after rehydration in water.

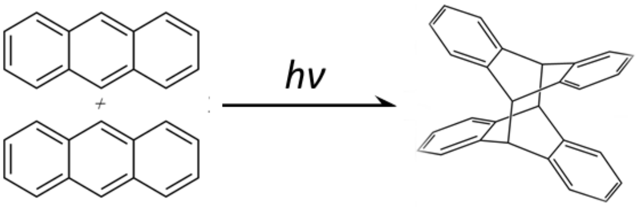

Fig. S5 Photodimerisation of two anthracene molecules.
